# Farnesoid X receptor promotes renal ischaemia‐reperfusion injury by inducing tubular epithelial cell apoptosis

**DOI:** 10.1111/cpr.13005

**Published:** 2021-02-16

**Authors:** Yao Xu, Dawei Li, Jiajin Wu, Minfang Zhang, Xinghua Shao, Longmei Xu, Lumin Tang, Minyan Zhu, Zhaohui Ni, Ming Zhang, Shan Mou

**Affiliations:** ^1^ Department of Nephrology School of Medicine, Renji Hospital Shanghai Jiaotong University Shanghai China; ^2^ Department of Urology School of Medicine, Renji Hospital Shanghai Jiaotong University Shanghai China

**Keywords:** acute kidney injury, apoptosis, farnesoid X receptor FXR, ischaemia‐reperfusion, renal tubular epithelial cells

## Abstract

**Purpose:**

We investigated the role of farnesoid X receptor (FXR), a ligand‐dependent transcription factor, in renal ischaemia‐reperfusion (I/R) injury.

**Materials and Methods:**

We performed unilateral renal I/R model in FXR knockout (*Fxr^−/−^*) and wild‐type (WT) mice in vivo and a hypoxia‐reoxygenation (H/R) model in vitro. The pathways by which FXR induces apoptosis were detected using a proteome profiler array. The effects of FXR on apoptosis were evaluated using immunoblotting, TUNEL assays and flow cytometry.

**Results:**

Compared with WT mice, *Fxr^−/−^* mice showed improved renal function and reduced tubular injury scores and apoptosis. Consistent with the in vivo results, the silencing of FXR decreased the number of apoptotic HK‐2 cells after H/R, while FXR overexpression aggravated apoptosis. Notably, bone marrow transplantation (BMT) and immunohistochemistry experiments revealed the involvement of FXR in the tubular epithelium rather than in inflammatory cells. Furthermore, in vivo and in vitro studies demonstrated that FXR deficiency increased phosphorylated Bcl‐2 agonist of cell death (p‐Bad) expression levels and the ratio of Bcl‐2/Bcl‐xL to Bax expression in the kidney. Treatment with wortmannin, which reduced p‐Bad expression, inhibited the effects of FXR deficiency and eliminated the tolerance of *Fxr^−/−^* mouse kidneys to I/R injury.

**Conclusions:**

These results established the pivotal importance of FXR inactivation in tubular epithelial cells after I/R injury. FXR may promote the apoptosis of renal tubular epithelial cells by inhibiting PI3k/Akt‐mediated Bad phosphorylation to cause renal I/R damage.

## Graphical abstract

We found the important role of FXR in survival signalling and death regulation in renal tubular epithelium, providing support for FXR as a vital regulator of renal biology. It suggested that FXR participated in renal I/R injury and could be a therapeutic target for AKI.

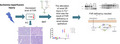

## 1 INTRODUCTION

Acute kidney injury (AKI), a sudden (within 48 hours) decline in renal function, is a common clinical emergency with high morbidity and mortality.^1^ As previously reported, AKI develops in more than 2%‐7% of hospitalized patients. In particular, in the intensive care unit, the prevalence of AKI is greater than 50%.^2^, ^3^ AKI can cause a series of complications, such as volume overload, hyperkalaemia, metabolic acidosis, uraemic complications and drug toxicity.^4^, ^5^ Despite advances in prevention and treatment, the prognosis after AKI has not significantly improved in the past decade.

Ischaemia‐reperfusion (I/R) injury is one of the leading causes of AKI. I/R generally induces significant tubular damage, which is characterized by cell flattening, tubular epithelium shedding, exposure of the underlying tubular basement membrane and luminal cast formation.^6^, ^7^ Therefore, it is of great clinical significance to understand the pathogenesis of renal I/R injury and to find effective preventative measures. Experimental animal models of renal I/R injury have been widely used to study the pathogenesis of ischaemic AKI.^1^, ^8^ In this study, we developed a unilateral I/R model with a consistent background to explore the pathophysiology of AKI.

Farnesoid X receptor (FXR), which was first found in the rat liver in 1995 by Forman, is a member of the nuclear receptor superfamily of ligand‐dependent transcription factors.^9^ It was named for its enhanced transcriptional activity in response to farnesol at concentrations above physiologic levels.^9^ FXR has been shown to be essential in various physiological processes, including bile acid metabolism, glucose metabolism and lipid metabolism.^10^, ^11^ Recent research has revealed that in addition to the liver, small intestine and adrenal glands, FXR is also expressed in adipose tissue, heart, spleen and kidney.^12^, ^13^, ^14^ Moreover, FXR plays an important role in the pathophysiological changes in blood vessels and participates in I/R injury of the small intestine, heart and other tissues.^12^, ^15^

The kidney expresses high levels of FXR. It has been reported that FXR has direct or indirect effects on the processes of kidney inflammation, oxidative stress, fibrosis and lipid metabolism.^16^ However, the potential function of FXR in the kidney remains largely unknown. In this study, we investigated FXR expression in kidney tissues. We report that FXR is expressed in the renal epithelium, is a novel apoptosis mediator and contributes to renal I/R injury.

## 2 MATERIALS AND METHODS

### 2.1 Animals

FXR knockout (*Fxr^−/−^*) mice (C57BL/6 background, stock number: 007 214) were purchased from the Jackson Laboratory (Bar Harbor, ME, USA). Male *Fxr^−/−^* mice and wild‐type (WT) mice (8‐12 weeks old and approximately 20‐25 g) were used in this study. Mice were raised in specific pathogen‐free conditions at 24 ± 1°C, 40 ± 1% humidity, a12 hours light/dark cycle, and with free access to food and water.

### 2.2 Renal I/R model and drug treatment

A warm renal I/R model was established as described.^17^, ^18^ The details of the operation and the treatment of pharmacological agents are described in the Supplementary Information.

All animal experiments were conducted following the NIH guidelines for the Care and Use of Laboratory Animals and the Animal Protocol Committee of Shanghai Jiaotong University and were approved by the Animal Care Committee at Renji Hospital, School of Medicine, Shanghai Jiaotong University.

### 2.3 Cell culture and treatment

The human proximal tubular cell line (HK‐2) was acquired from the American Type Culture Collection (ATCC; Manassas, VA, USA). The details of the cell culture and the treatment of pharmacological agents are described in the Supplementary Information.

### 2.4 Renal function, survival and histomorphological analyses

Plasma creatinine (Cr) and urea nitrogen (BUN) levels were measured with a standard spectrophotometric assay (Roche Diagnostic GmbH, Germany). The Kaplan‐Meier survival analytical method was used to estimate the survival rate and to generate a survival curve for the mice. The kidneys were harvested for periodic acid‐Schiff (PAS) staining and myeloperoxidase (MPO) staining, or subjected to the terminal deoxynucleotidyl transferase‐mediated 2′ deoxyuridine 5′‐triphosphate nick‐end labelling (TUNEL) assay, as previously described.^18^, ^19^ The immunohistochemical localization of FXR in renal sections was determined using an NR1H4 antibody (1:200, #A9003A; R&D Systems, USA). Details are provided in the Supplementary Information.

### 2.5 RNA sequencing (RNA‐seq) and the identification of differentially expressed transcripts

Kidney tissues were sent to the Genminix Biological Company (Shanghai, China) for microarray analysis. Details are provided in the Supplementary Information.

### 2.6 Mouse apoptosis proteome profiler array

To investigate the pathways by which FXR induces apoptosis, we examined apoptosis‐related proteins using a proteome profiler array. Details are provided in the Supplementary Information.

### 2.7 Bone marrow transplantation (BMT)

BMT was performed as previously described (Figure S1).^17^, ^19^ The details of BMT are described in the Supplementary Information. Renal I/R procedures were conducted 30 days after BMT.

### 2.8 Transcriptional analysis and Western blot (WB) analysis

Kidney tissues or HK‐2 cells were subjected to transcriptional or WB analyses. Experimental procedures, primer sequences and antibody information are described in the Supplementary Information.

### 2.9 Small interfering RNA (siRNA)

siRNA duplexes targeting FXR, as well as non‐targeted scrambled siRNA duplexes, were provided by Invitrogen (Life Technologies Corporation, NY, USA). The details of RNA interference and siRNA sequences are described in the Supplementary Information.

### 2.10 Fluorescence‐activated cell sorting (FACS) analysis

Flow cytometry was used to analyse apoptosis after H/R. Details are provided in the Supplementary Information.

### 2.11 Polymerase chain reaction (PCR) genotyping

Routine PCR genotyping was performed to confirm the knockout allele in *Fxr^−/−^* mice. DNA was extracted from the tails of mice. Primer sequences were as follows: wild‐type forward: TCTCTTTAAGTGATGACGGGAATCT; mutant forward: GCTCTAAGGAGAGTCACTTGTGCA; and common: GCATGCTCTGTTCATAAACGCCAT. These primers produced fragments of 291 bp in *Fxr^–/–^* tissues. DNA from the tail of a wild‐type mouse was 249 bp.

### 2.12 Plasmid transfection

A plasmid for overexpression of FXR under the CMV was constructed by Genomeditech (Shanghai, China). The HK‐2 cells were transfected with plasmid (3 μg) for 12 hours using Lipofectamine™ 3000 Transfection Reagent (Thermo Fisher Scientific, L3000150) according to the manufacturer's instructions.

### 2.13 Statistics

All values are expressed as the mean ± the standard deviation of the mean (SD). Differences between two parameters were analysed by an unpaired Student's *t* test. Statistical significance was set at *P* < 0.05.

## 3 RESULTS

### 3.1 Renal expression of FXR protein is upregulated after renal I/R

**FIGURE 1:**
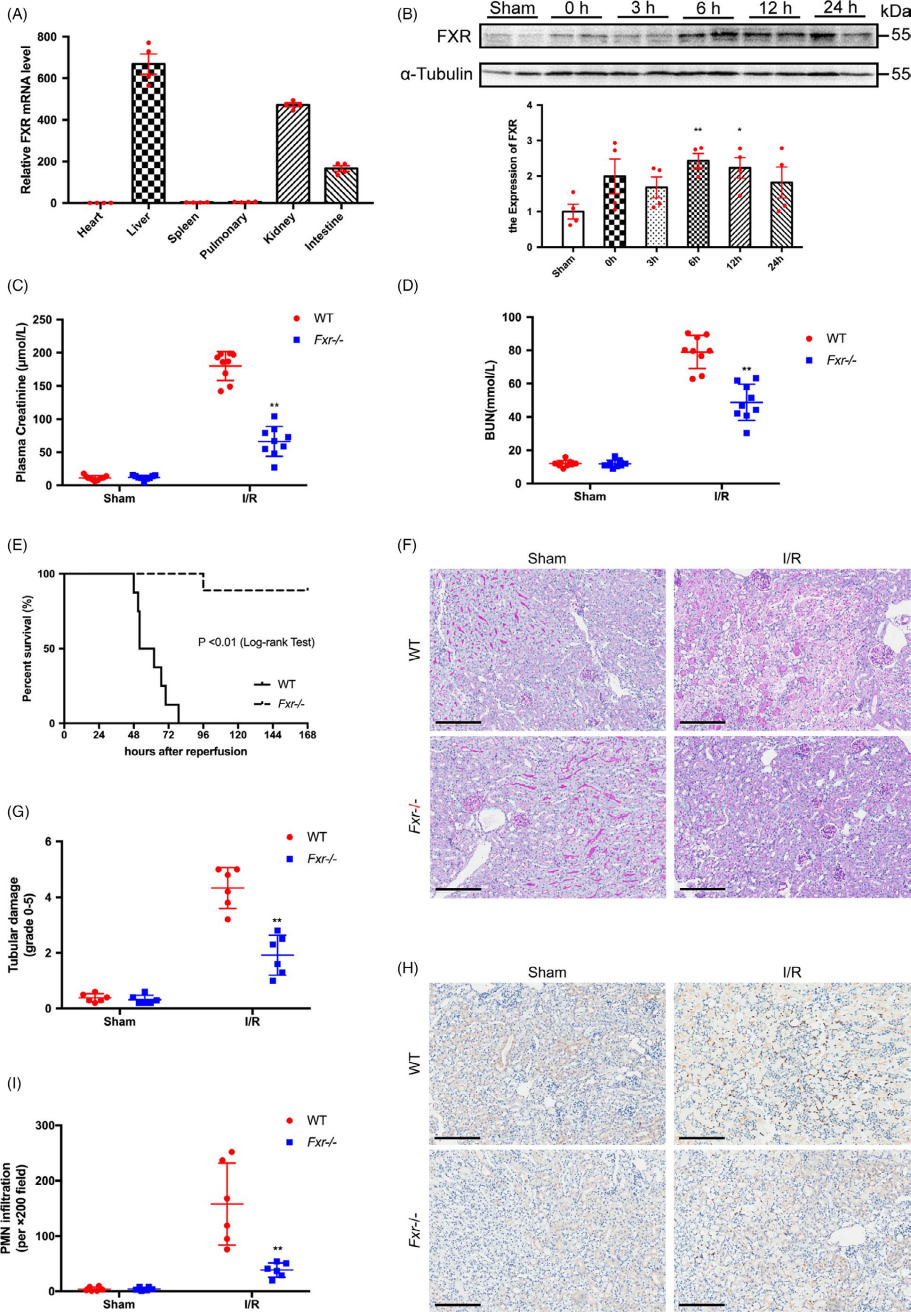
FXR deficiency alleviates kidney injury after I/R. (A) FXR is expressed in the kidney. Total RNA was extracted from the indicated tissues of wild‐type mice, and FXR mRNA levels were measured by RT‐PCR. Results are plotted as the fold change relative to mRNA levels in heart (n = 4). (B) Time courses of FXR protein expression in the kidney after I/R were measured by WB. Whole cell lysates of the kidney tissues at 0 h, 3 h, 6 h, 12 h and 24 h after reperfusion were analysed (n = 4). Plasma creatinine (C) and BUN (D) concentrations were measured at 24 h after the initiation of reperfusion in wild‐type (WT) and *Fxr^−/−^* mice (n = 9 per group). (E) Kaplan‐Meier Estimates of survival among wild‐type and *Fxr^−/−^* mice (n = 20 per group) after renal I/R injury (log‐rank test, *P* < 0.01). (F) PAS staining and (G) quantitative data of the tubular injury score of post‐I/R kidneys harvested at 24 h (original magnification, ×200; scale bar, 200 μm) (n = 6 per group). (H) Representative renal myeloperoxidase (MPO) staining (original magnification, ×200; scale bar, 200 μm) (n = 6 per group). (I) The number of infiltrating MPO‐positive cells in the post‐I/R kidney. Each column represents the mean ± SD. *P < 0.05 and **P < 0.01 vs sham (B) or wild‐type mice at the same time point after I/R injury (C‐I)

RT‐PCR was used to detect FXR mRNA concentrations in the heart, liver, spleen, lungs, kidney and intestine. The highest FXR levels were in the liver. Low levels of FXR mRNA were detected in the heart, spleen and lungs. FXR mRNA was also highly expressed in the kidney, which was consistent with previous findings (Figure 1A).^14^

Previous studies have reported that renal I/R induces acute kidney disease.^6^, ^20^ Next, we established a renal I/R model. At different time points after the initiation of reperfusion following 20 minutes of ischaemia, the expression of FXR protein was analysed. FXR protein expression was increased after renal ischaemia by reperfusion at every point after ischaemia, demonstrating the highest expression at 6 hours after reperfusion (Figure 1B). Time courses of renal FXR mRNA expression after I/R are shown in the Supplementary Information (Figure S2).

### 3.2 FXR deficiency alleviates kidney injury after I/R

To determine whether FXR was involved in renal I/R injury, we generated a renal I/R model in *Fxr^−/−^* and WT mice. Successful generation of *Fxr^−/−^* mice was confirmed by PCR genotyping (Figure S3A), and no enzyme expression was detected in *Fxr^−/−^* mice by WB analyses (Figure S3B‐C). We tested the renal function of the two groups of mice at 24 hours after reperfusion following 20 minutes of ischaemia. As shown in Figure 1C and 1D, *Fxr^−/−^* mice had less kidney dysfunction with significantly lower levels of Cr and BUN (*P* < 0.01).

Histological evidence based on PAS staining reinforced this observation (Figure 1F and 1G). Compared with mice that received the sham operation, WT mice subjected to I/R exhibited marked tubular injury, especially in corticomedullary junctions, which included extensive necrosis of tubular epithelial cells, loss of the brush border, exposure of the basement membrane, tubular dilation, intratubular cell debris and cast formation. A large number of infiltrating inflammatory cells were found in the interstitium. The pathological changes were significantly reduced in *Fxr^−/−^* mice subjected to I/R, with only a subset of tubular epithelial cells vacuolated and a small number of inflammatory cells in the interstitium. The semiquantitative assessment of kidney injury revealed tubular necrosis scores of 4.33 ± 0.30 and 1.92 ± 0.29, respectively, in WT and *Fxr^−/−^* mice at 24 hours after I/R injury. Likewise, *Fxr^−/−^* mice exhibited significantly less neutrophil infiltration after reperfusion compared with WT mice based on MPO staining (Figure 1H,I).

We further recorded the survival of *Fxr^−/−^* and WT mice for 7 days after reperfusion following 25 minutes of ischaemia (Figure 1E). Between 48 and 96 hours after renal I/R injury, all WT mice died, while most *Fxr^−/−^* mice survived (90%, *P* < 0.01). These data suggested that knockout of FXR protected mice from renal I/R injury.

### 3.3 FXR deficiency attenuates I/R‐induced apoptosis

**FIGURE 2:**
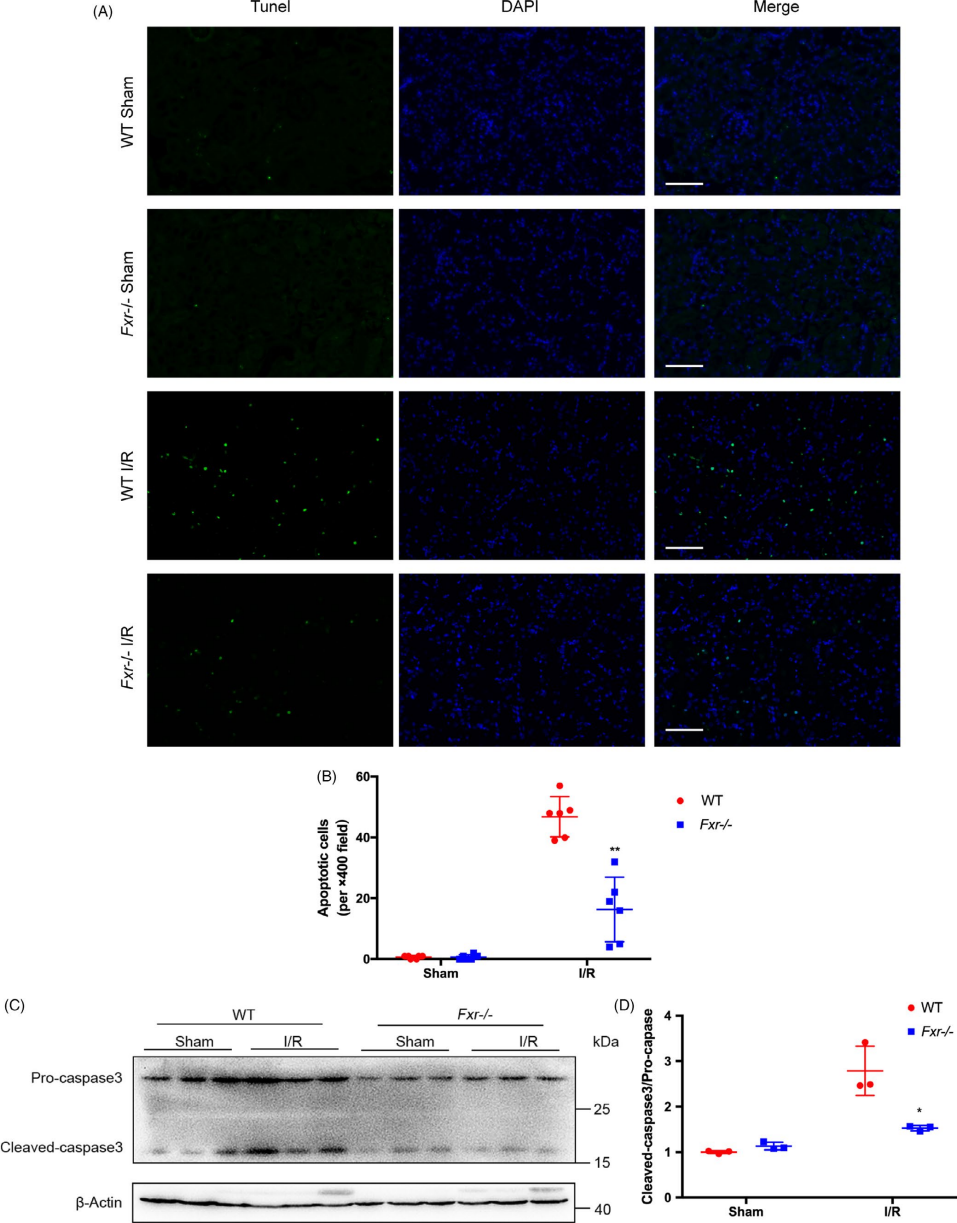
FXR deficiency attenuates I/R‐induced apoptosis. Kidneys were harvested at 24 h reperfusion after 20 min ischaemia in mice. (A) Representative images of TUNEL (terminal deoxynucleotidyl transferase dUTP nick‐end labelling) assays (original magnification, ×400; scale bar, 100 μm). Left, TUNEL staining; middle 4',6‐diamidino‐2‐ phenylindole (DAPI)–stained nucleus; right, TUNEL overlaps with DAPI. (B) Quantitative analysis of apoptosis cells (n = 6 per group). (C) Representative WB images of caspase‐3 in renal tissue 24 h post‐I/R (n = 3 per group). (D) Expression ratio of cleaved caspase‐3 to pro‐caspase‐3 at 24 h post‐I/R. The densities of caspase‐3 protein bands were quantified with ImageJ analytical software and normalized to β‐actin. Each column represents the mean ± SD. *P < 0.05 vs wild‐type mice at the same time point after I/R injury

We assessed the role of FXR in renal I/R‐triggered apoptosis by a TUNEL assay. As shown in Figure 2, TUNEL‐positive tubular cells were hardly detectable in the kidney tissue of WT and *Fxr^−/−^* mice subjected to the sham surgery. I/R induced significant tubular apoptosis in the kidneys of WT mice and, to a lesser extent, in *Fxr^−/−^* mice following 20 minutes of ischaemia and 24 hours of reperfusion (*P* < 0.01, Figure 2A,B).

Caspase‐3, a caspase family member, is involved in the downstream effects of various apoptosis pathways. It is called the ‘death execution protease’ and is the most important end‐shear enzyme in the process of apoptosis.^21^ Consistent with the results of the TUNEL assay, *Fxr^−/−^* mice exhibited a 2‐fold decrease in caspase‐3 expression at the protein level compared with WT mice after I/R (Figure 2C,D).

**FIGURE 3:**
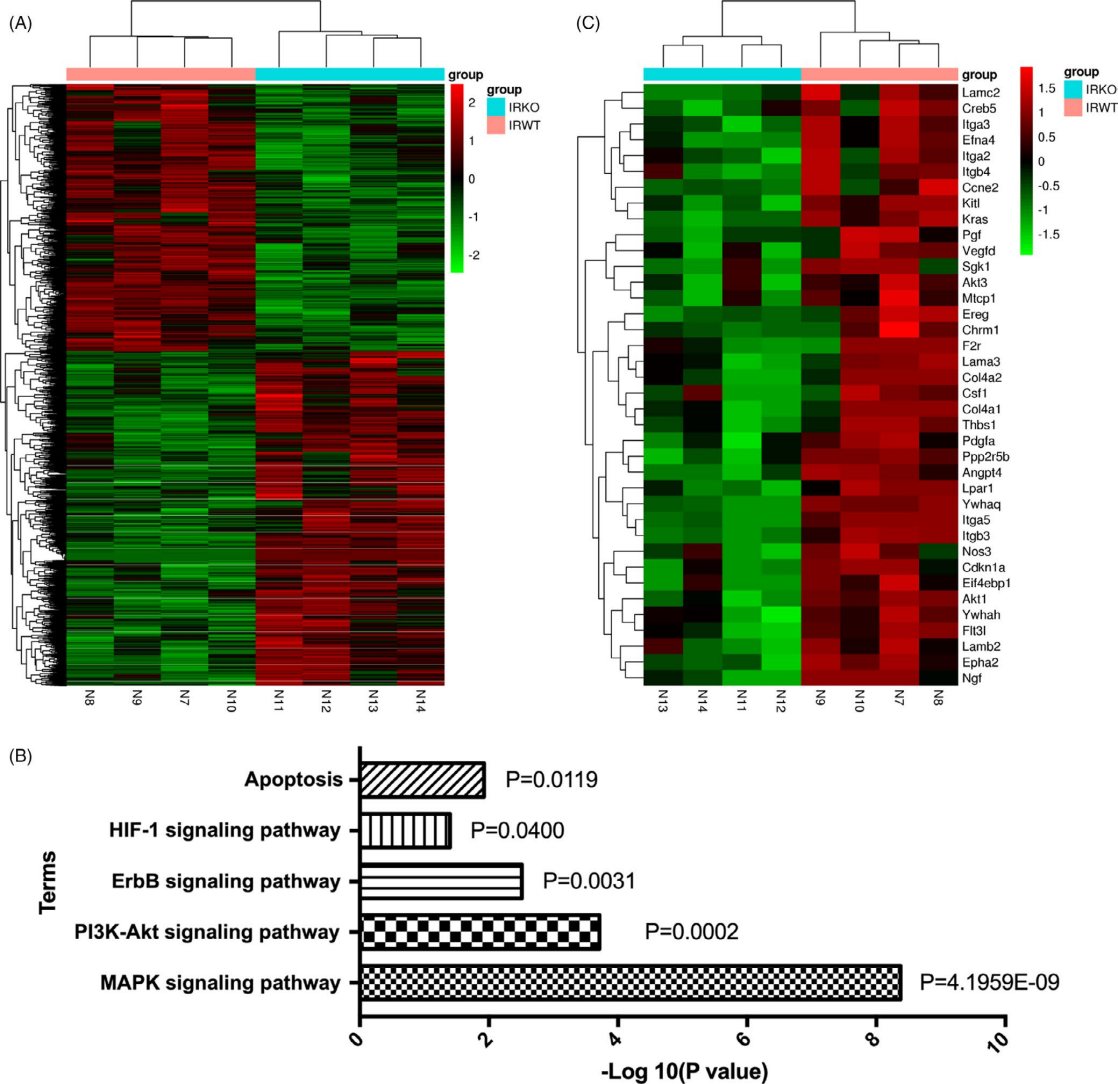
Gene expression patterns and signalling pathways associated with FXR knockout in renal I/R injury. RNA‐seq analysis was used to reveal the DEGs between wild‐type and *Fxr^−/−^* mice (n = 4 per group). (A) Cluster analysis of DEGs between two groups. Red means upregulated and green means downregulated. (B) The five most enriched KEGG pathways between wild‐type and *Fxr^−/−^* mice. (C) Cluster analysis of DEGs involved in the most enriched KEGG pathways

To clarify the molecular mechanisms by which FXR knockout alleviated I/R injury, we conducted a whole‐genome RNA‐seq analysis in renal total RNA isolated from post‐I/R WT and *Fxr^−/−^* mice. Four cDNA libraries constructed from each group were sequenced, and a total of 225 676 670 single‐end reads were generated. After removing adaptor sequences and low‐quality reads, 221 188 332 reads were mapped to the reference genome of mice using HISAT2. With a threshold of a log2 (fold change) cut‐off value > 1.2 and *P* < 0.05, differential expression analysis identified 3228 genes with significant expression changes, which included 1795 upregulated and 1433 downregulated differentially expressed genes (DEGs) (Figure 3A). The Kyoto Encyclopedia of Genes and Genomes (KEGG) pathway enrichment analysis was used to examine the biological attributes of FXR‐driven genes in I/R after excluding genes shared with controls. As shown in Figure 3B, we found that apoptosis, the HIF‐1 signalling pathway, the ErbB signalling pathway, the PI3K‐Akt signalling pathway and the MAPK signalling pathway were the five most enriched KEGG pathways. Strikingly, these pathways contributed to apoptosis. Heat maps of the DEGs involved in these pathways are depicted in Figure 3C. In conclusion, RNA‐seq revealed that FXR‐mediated apoptosis contributed to the onset of I/R injury.

### 3.4 FXR deficiency promotes post‐I/R phosphorylation of Bad

**FIGURE 4:**
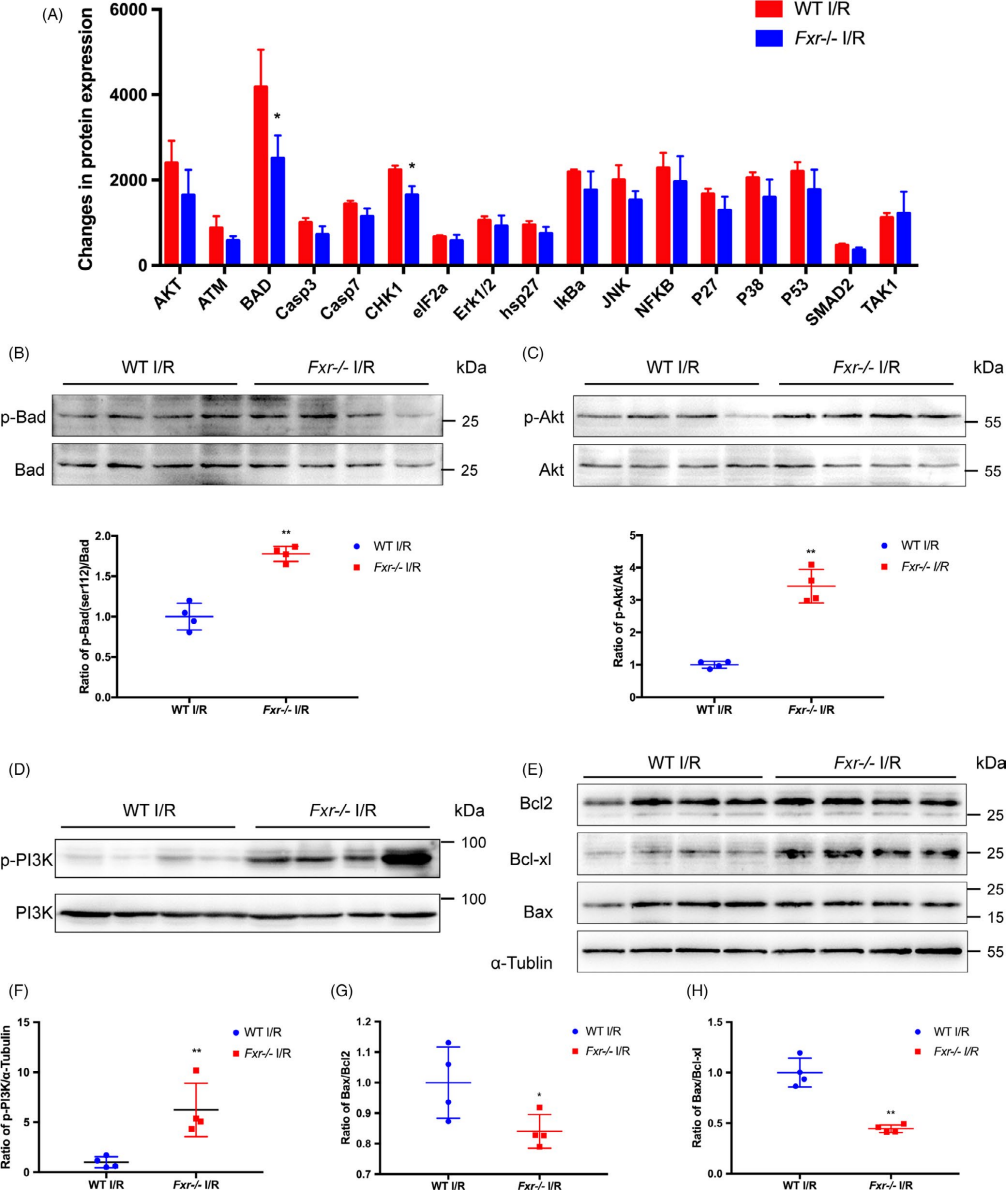
Expression of Bad signalling pathway–related proteins in renal I/R injury. Wild‐type and *Fxr^−/−^* mice were subjected to 20 min of renal I/R, and kidneys were harvested at 24 h after surgery. (A) Quantitative analysis of the mouse apoptosis signalling pathway array in post‐I/R kidney. Histogram of quantitative analysis showing the different protein expression of the apoptotic markers between wild‐type and *Fxr^−/−^* mice. (B) Representative WB images and expression ratio of p‐Bad to t‐Bad. (C) Representative WB images and expression ratio of p‐Akt to t‐Akt. (D) Representative WB images and (F) expression of p‐PI3K. (E) Representative WB images of Bcl‐2, Bcl‐xL and Bax. (G) Expression ratio of Bax to Bcl‐2. (H) Expression ratio of Bax to Bcl‐xL. The densities of bands were quantified with ImageJ analytical software. Each column represents the mean ± SD. *P < 0.05 and **P < 0.01 vs wild‐type mice at the same time point after I/R injury

To further determine the mechanism of apoptosis induced by FXR in I/R, we used a Mouse Apoptosis Signaling Pathway Array Kit. After exposure to 24 hours of reperfusion, the kidneys were harvested, and apoptotic markers were examined using the array. In Figure S4A‐B, the images show the changes in apoptotic markers in WT and *Fxr^−/−^* mice. Based on semiquantitative analysis, the marker with the most significant difference in expression between WT and *Fxr^−/−^* mice involved in the apoptosis signalling pathway was Bad (Figure 4A).

In an effort to identify whether kidney injury induced by FXR was associated with the activation of proapoptotic Bad signalling, we performed WB to detect the expression of Bad signalling pathway–related proteins in post‐I/R kidneys. After 24 h of reperfusion, the total Bad expression level decreased, and the ratio of p‐Bad to Bad expression was significantly higher in *Fxr^−/−^* than WT mouse kidneys (Figure 4B), indicating that knockout of FXR activates the phosphorylation of Bad. We also examined the expression changes in the PI3K‐Akt signalling pathway. Akt is a regulatory protein upstream of Bad.^22^ Strikingly, after reperfusion, the level of p‐Akt was also significantly higher in *Fxr^−/−^* mouse kidneys than in WT mouse kidneys, indicating that the knockout of FXR augmented post‐IR activation of Akt signalling (Figure 4C). The expression of phosphorylated‐PI3K was also significantly higher in *Fxr^−/−^* than WT mouse kidneys (Figure 4D,F).

Consistent with the increases in Bad activation, following reperfusion, the levels of Bcl‐2 and Bcl‐xL, Bcl‐2 family proteins that repress apoptosis, were higher in *Fxr^−/−^* than WT mouse kidneys. Compared with WT mice, after I/R, Bax expression decreased in *Fxr^−/−^* mice, resulting in a significant reduction in the ratio of Bax to Bcl‐2 and Bcl‐xl in *Fxr^−/−^* mouse kidneys (Figure 4E, G and H).

### 3.5 FXR deficiency in renal tubular epithelial cells alleviates renal I/R injury

**FIGURE 5:**
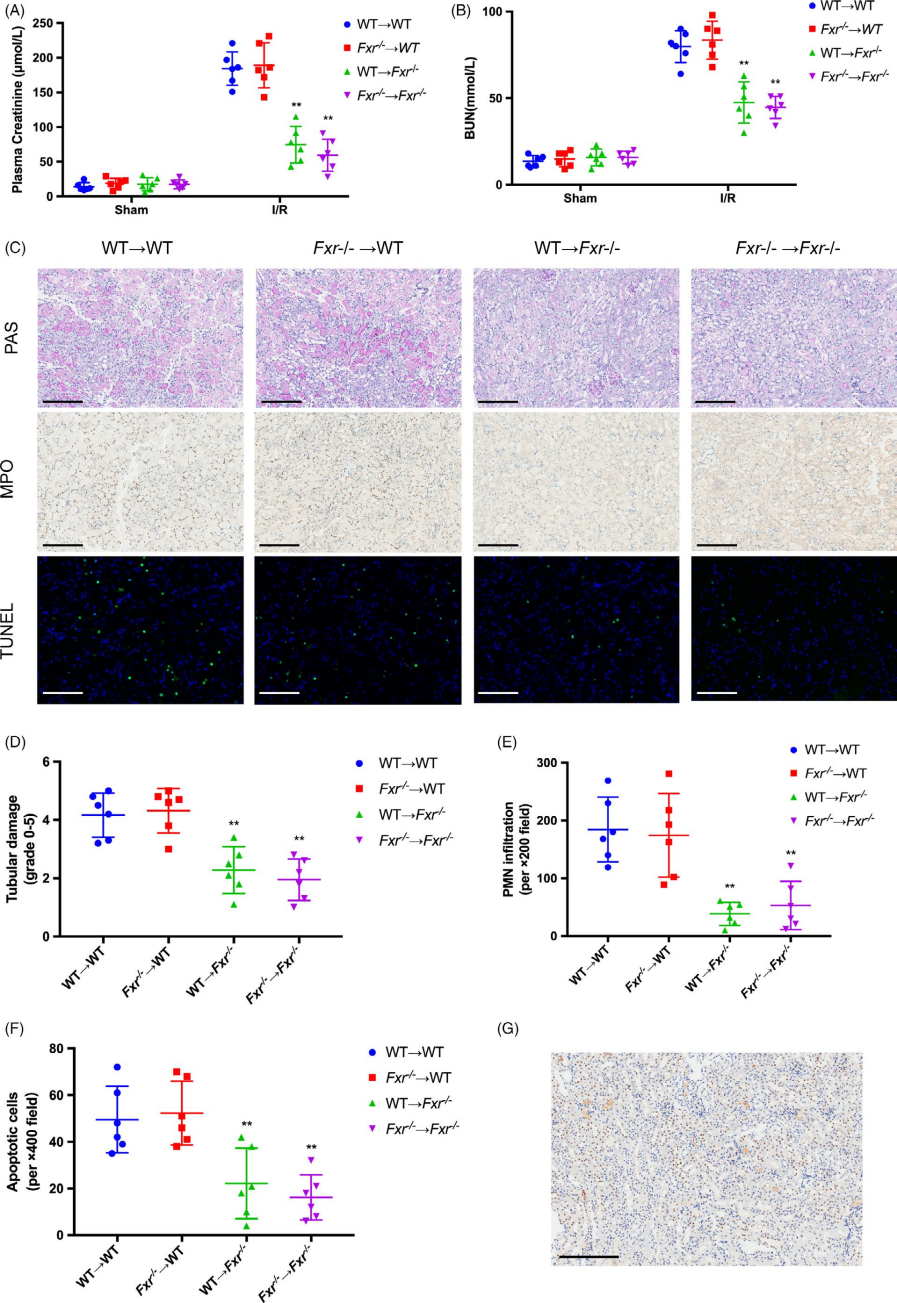
Contribution of FXR in inflammatory cells or renal parenchymal cells in renal I/R injury. Bone marrow transplantation (WT → *Fxr^−/−^*, *Fxr^−/−^*→ *Fxr^−/−^*, WT → *Fxr^−/−^* and *Fxr^−/−^*→ *Fxr^−/−^*) was performed 30 days before the renal I/R procedures (n = 9 per group). Kidneys were harvested after 24 h of reperfusion and 20 min of ischaemia in mice. Plasma creatinine (A) and BUN (B) concentrations were measured. (C) PAS staining (original magnification, ×200; scale bar, 200 μm), myeloperoxidase (MPO) staining (original magnification, ×200; scale bar, 200 μm) and TUNEL staining (original magnification, ×400; scale bar, 100 μm). (D) Quantitative data of the tubular injury score. (E) The number of infiltrating MPO‐positive cells. (F) Quantitative analysis of apoptosis cells. (G) Immunohistochemistry of FXR in renal sections. Each column represents the mean ± SD. *P < 0.05, ** *P* < 0.01

Although the final result of renal I/R injury was renal parenchymal cell death, the severity of the injury depended in part on the inflammatory response. We performed BMT to investigate whether FXR induction in inflammatory cells was involved in renal I/R injury (Figure S1). WT mice were lethally irradiated, and the bone marrow was reconstituted with bone marrow from *Fxr^−/−^* mice (*Fxr^−/−^*→ WT) or WT mice (WT → WT). Using a similar method, we created WT → *Fxr^−/−^* or *Fxr^−/−^*→ *Fxr^−/−^* mice. After 30 days, the mice were subjected to I/R procedures. After 24 hours of reperfusion following 20 minutes of ischaemia, there was a marked increase in plasma Cr (Figure 5A) and BUN (Figure 5B) in WT → WT and *Fxr^−/−^*→ WT mice, but not in WT → *Fxr^−/−^* and *Fxr^−/−^*→ *Fxr^−/−^* mice. Kidneys of WT → *Fxr^−/−^* and *Fxr^−/−^*→ *Fxr^−/−^* mice had milder tubular injuries (Figure 5C) and lower tubular necrosis scores (Figure 5D) after I/R based on PAS staining. The infiltration of neutrophils increased in WT → WT and *Fxr^−/−^*→ WT mice after I/R compared with WT → *Fxr^−/−^* and *Fxr^−/−^*→ *Fxr^−/−^* mice (Figure 5C,E). Likewise, more TUNEL‐positive cells were detectable in WT → *Fxr^−/−^* and *Fxr^−/−^*→ *Fxr^−/−^* mice (Figure 5C,F). These results indicated that FXR expression in inflammatory cells did not play a role in this setting. The immunohistochemical analysis of FXR in renal tissue showed that FXR was mainly expressed in renal tubular epithelial cells (Figure 5G). Therefore, we speculated that the alleviation of renal I/R injury in *Fxr^−/−^* mice was caused by a lack of FXR in renal tubular epithelial cells.

### 3.6 Silencing of FXR activates the phosphorylation of Bad and decreases the apoptosis of renal tubular epithelial cells

**FIGURE 6:**
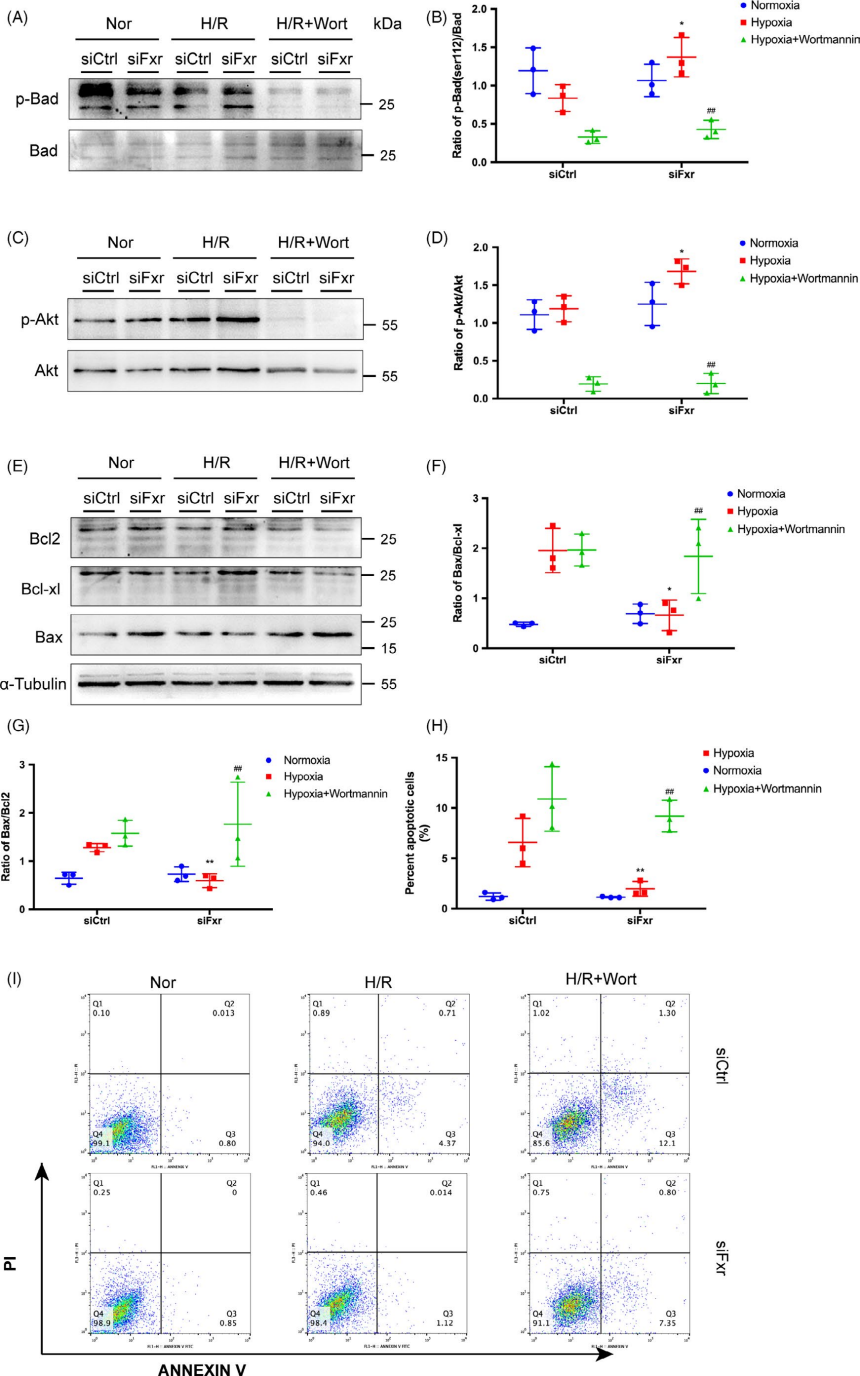
Inhibition of Bad phosphorylation by wortmannin, an inhibitor of PI3K, aggravates the apoptosis of renal tubular epithelial cells. HK‐2 cells were transfected with FXR siRNA (siFxr) or control siRNA (siCtrl). Cells were treated with wortmannin (Wort) or not and then exposed to hypoxia (1% O2) for 24 h followed by 6 h of reoxygenation. (A) Representative WB images and (B) expression ratio of p‐Bad to t‐Bad. (C) Representative WB images and (D) expression ratio of p‐Akt to t‐Akt. (E‐G) Representative WB images of Bcl‐2, Bcl‐xL and Bax; left: expression ratio of Bax to Bcl‐2; right: expression ratio of Bax to Bcl‐xL. (H) and (I) H/R‐induced apoptotic cells were counted by Flow cytometry. The densities of bands were quantified with ImageJ analytical software. Each column represents the mean ± SD. *P < 0.05 vs siCtrl after H/R, ^##^ *P* < 0.01 vs siFxr after H/R

To determine the role of FXR in renal tubular epithelial cells, we knocked down FXR expression with specific siRNAs before these HK‐2 cells were subjected to H/R. Compared with control siRNA‐transfected cells, the cells transfected with FXR siRNA showed increased p‐Bad expression after H/R (Figure 6A,B). Consistent with the changes in vivo, after H/R, the level of p‐Akt was significantly higher in FXR siRNA‐transfected cells than in control siRNA‐transfected cells (Figure 6C,D). H/R resulted in an increase in the ratio of Bax to Bcl‐2 and Bcl‐xL in the controls but not in the FXR‐silenced cells (Figure 6E‐G).

To clarify whether the protection observed in HK‐2 cells resulted from increased phosphorylation of Bad, we treated cells with wortmannin, an inhibitor of PI3K, before H/R, to inhibit Akt phosphorylation, further inhibiting Bad phosphorylation. After H/R, the levels of p‐Bad and p‐Akt in wortmannin‐treated cells were significantly lower than those in the vehicle‐treated cells (Figure 6A‐D), showing that wortmannin treatment inhibited the Bad and Akt activation induced by FXR silencing. The ratio of Bax to Bcl‐2 and Bcl‐xL in wortmannin‐treated siFXR HK‐2 cells was significantly higher than that in vehicle‐treated siFXR HK‐2 cells after H/R.

Flow cytometry confirmed that H/R‐induced apoptosis decreased significantly in FXR‐silenced cells compared with control siRNA‐transfected cells. However, after wortmannin treatment, there was no significant difference between FXR siRNA cells and control siRNA cells (*P* > 0.05). The results indicated that FXR was involved in H/R‐induced epithelial cell apoptosis (Figure 6H,I).

### 3.7 Overexpression of FXR contributes to apoptosis of renal tubular epithelial cells

**FIGURE 7:**
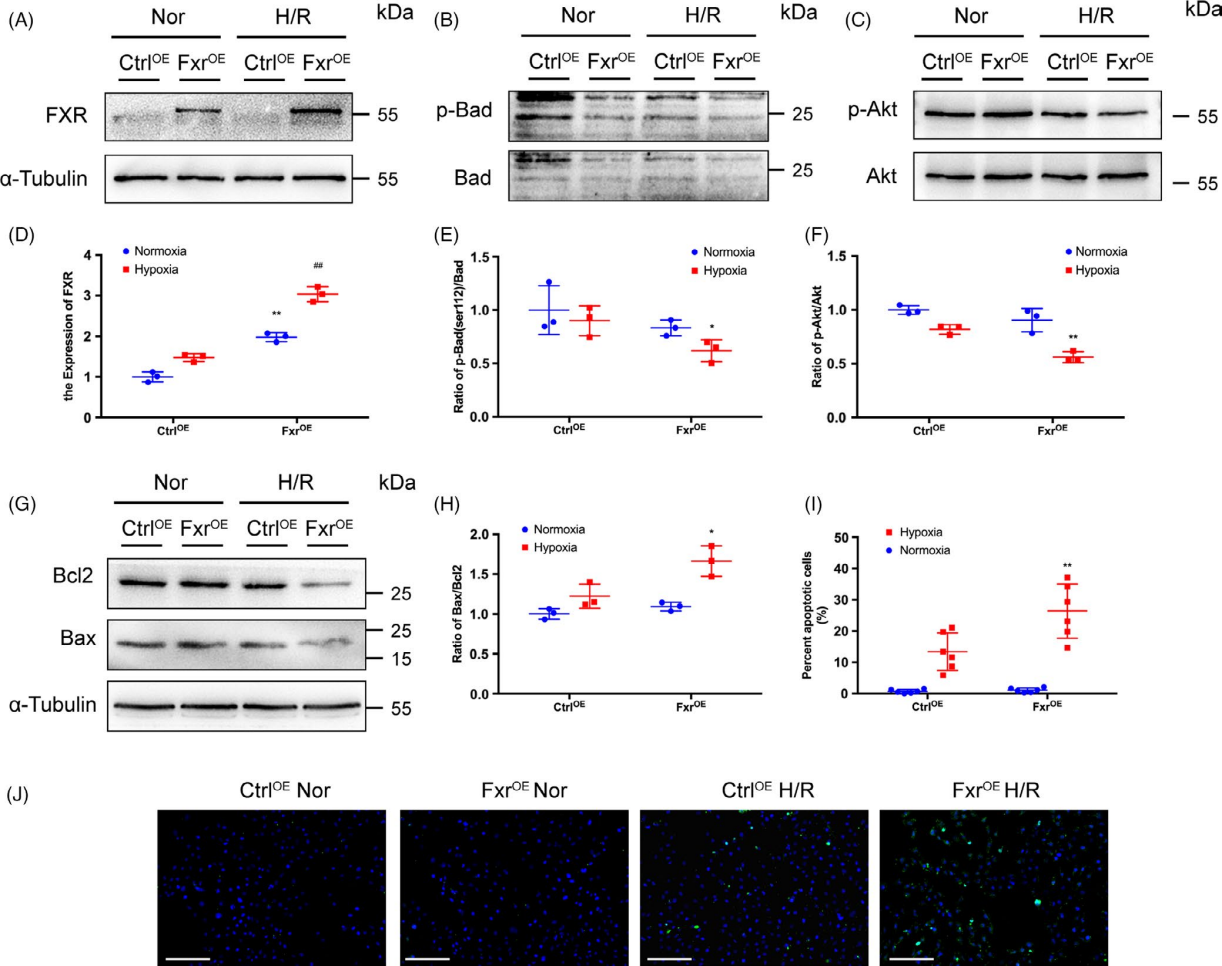
Overexpression of FXR in HK‐2 cells leads to increased cell apoptosis. HK‐2 cells were transfected with FXR plasmid (FXR^OE^) or empty vector (Ctrl^OE^). Cells were treated with wortmannin (Wort) or not and then exposed to hypoxia (1% O_2_) for 6 h followed by 3 h of reoxygenation. (A) Representative WB images and (D) expression of FXR (**P < 0.01 vs Ctrl^OE^ under normoxia, ^##^ *P* < 0.01 vs Ctrl^OE^ after H/R). (B) Representative WB images and (E) expression ratio of p‐Bad to t‐Bad. (C) Representative WB images and (F) expression ratio of p‐Akt to t‐Akt. (G) Representative WB images and (H) expression ratio of Bax to Bcl‐2. (I) Representative images of TUNEL assays (original magnification, ×200; scale bar, 200 μm). (J) Quantitative analysis of apoptosis cells (n = 6 per group). Each column represents the mean ± SD. *P < 0.05 vs siCtrl after H/R, **P < 0.01 vs Ctrl^OE^ after H/R

To further elucidate the role of FXR in renal I/R injury, the effect of FXR overexpression on H/R injury was determined. We used a plasmid to overexpress FXR under control of the cytomegalovirus (CMV) promoter in HK‐2 cells. After transfection, the cells were subjected to H/R. FXR‐overexpressing cells were successfully generated, which was verified by WB analysis (Figure 7A,D). Compared with cells transfected with the control vector, HK‐2 cells overexpressing FXR showed decreased p‐Bad expression after H/R and p‐Akt levels (Figure 7B‐C and E‐F). The ratio of Bax to Bcl‐2 was higher in FXR‐overexpressing cells than in control cells (Figure 7G,H). FXR overexpression resulted in a significantly increased number of TUNEL‐positive cells after H/R (Figure 7I,J).

### 3.8 Inactivation of Bad by wortmannin reduces the protection conferred by FXR knockout against I/R injury

**FIGURE 8:**
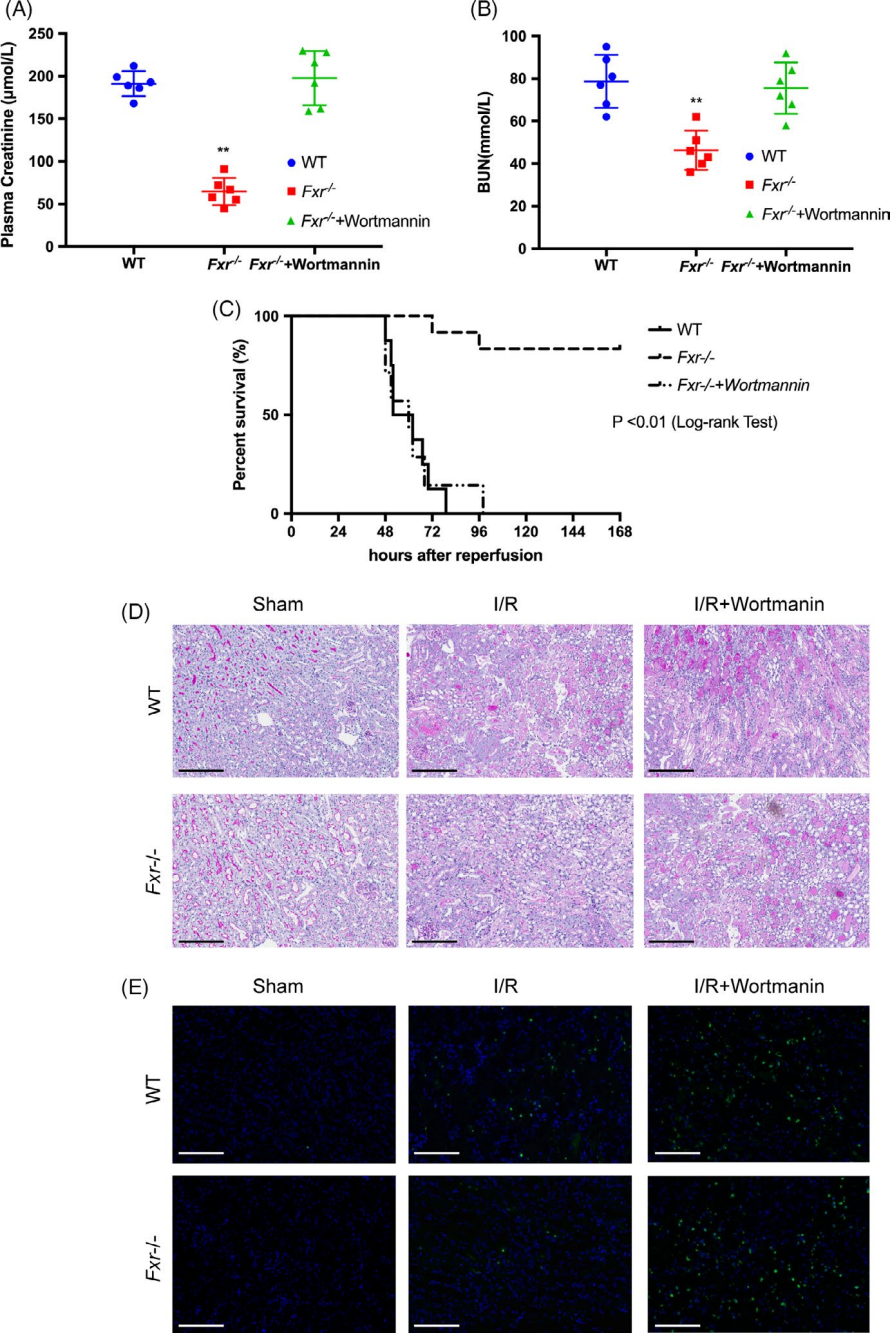
Inactivation of Bad by wortmannin reduces the protection conferred by FXR knockout against I/R injury. Wortmannin was administered to mice, and the mice were subjected to 20 min of renal ischaemia (I/R). Kidneys were harvested 24 h after surgery. Plasma creatinine (A) and BUN (B) concentrations were measured at 24 h after the initiation of reperfusion. (C) Kaplan‐Meier estimates of survival after renal I/R injury (log‐rank test, *P* < 0.01 *Fxr^−/−^* mice vs wild‐type mice). (D) PAS staining of post‐I/R kidneys harvested at 24 h (original magnification, ×200; scale bar, 200 μm). (E) Representative images of TUNEL assays (original magnification, ×400; scale bar, 100 μm). Each column represents the mean ± SD. *P < 0.05 and **P < 0.01 vs wild‐type mice

Twenty‐four hours after reperfusion, the levels of Cr and BUN were significantly higher in wortmannin‐treated than vehicle‐treated *Fxr^−/−^* mice (Figure 8A,B), showing that wortmannin treatment inhibited the protection induced by FXR knockout. The survival observations and histological manifestations were consistent with the serum results (Figure 8C,D). The number of TUNEL‐positive cells in wortmannin‐treated *Fxr^−/−^* mouse kidneys was significantly greater than in vehicle‐treated *Fxr^−/−^* kidneys after I/R, indicating that the administration of wortmannin abolished the anti‐apoptotic effects of FXR deficiency (Figure 8E).

## 4 DISCUSSION

**FIGURE 9:**
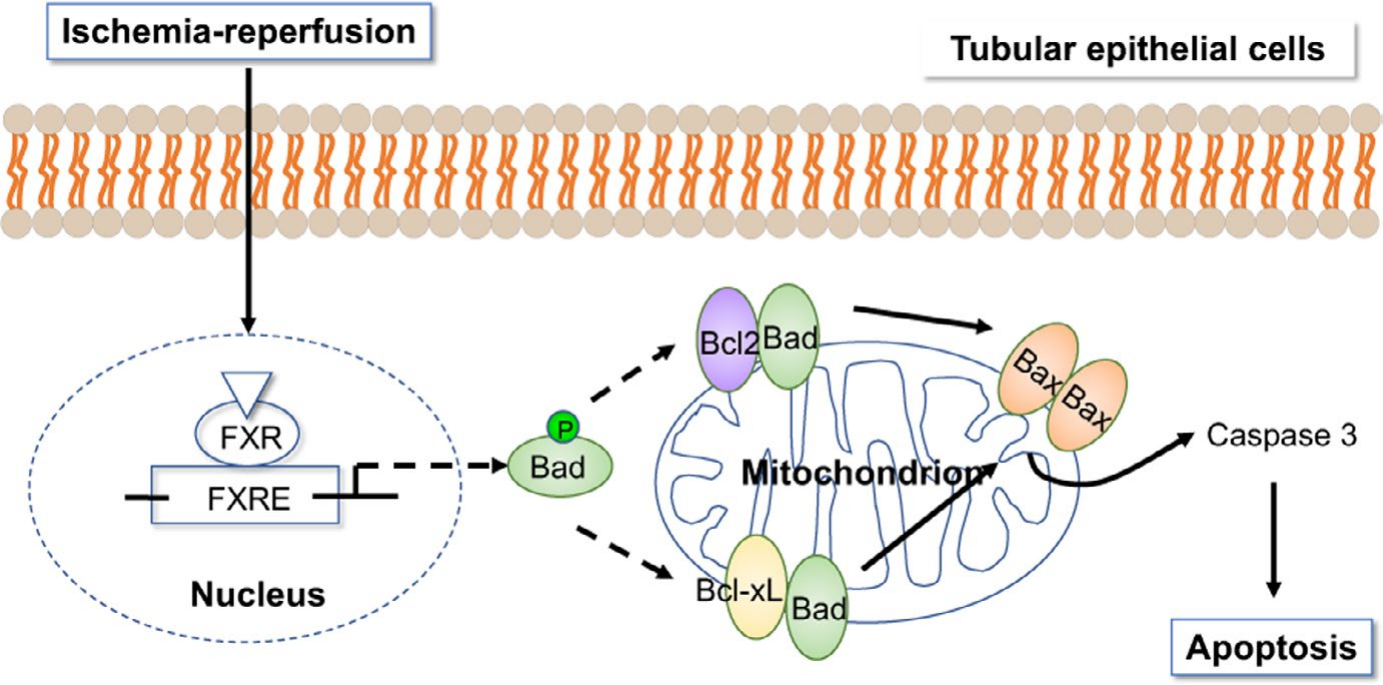
Schematic representation of FXR‐induced renal I/R injury. The insufficiency of FXR facilitated Bad phosphorylation, isolating Bad from the Bcl‐2/Bcl‐xL complex and leading to less apoptosis after I/R

In this study, we demonstrated that (i) FXR is expressed in the kidney and that FXR deficiency protects the kidney from I/R‐induced renal function impairment and apoptosis. (ii) This protection was a result of FXR deficiency in renal tubular epithelial cells but not in inflammatory cells. (iii) FXR silencing in epithelial cells activates the phosphorylation of Bad and decreases apoptosis while FXR overexpression enhances apoptosis, and (iv) the inhibition of Bad phosphorylation by wortmannin weakens the FXR deficiency–mediated renal protection and leads to an increase in apoptotic tubular epithelial cell death (Figure 9). These results indicate that kidney I/R injury induced by FXR is associated with an increase in apoptotic tubular epithelial cell death that results from repressing the phosphorylation of Bad.

FXR is a transcription factor that participates in the biosynthesis and enterohepatic circulation of bile acids. It has previously been shown that FXR plays an important role in I/R injury. Ferrigno et al^23^ found that the FXR agonist obeticholic acid (OCA) significantly reduces protein methyltransferase (PRMT‐1) and iNOS expression in post‐I/R livers, and it increases eNOS expression. Although there was no marked change in histological hepatocyte damage, OCA administration decreased hepatic serum enzyme and total bilirubin levels after I/R. It has also been reported that FXR expression levels are clearly decreased in liver after intestinal I/R.^15^ Pre‐treatment with OCA before intestinal I/R alleviates inflammation, protects intestinal barrier function and improves survival.^24^, ^25^ In contrast, FXR was shown to aggravate heart I/R damage. Pu et al^12^ found that FXR is expressed in various cardiac cells that contribute to myocardial I/R injury. Pharmacological inhibition or gene knockout of FXR significantly reduces the infarct area and improves cardiac function after I/R. Similarly, our results showed improved renal function, decreased tubular injury scores and extended survival in *Fxr^−/−^* compared with WT mice after I/R. A hallmark of renal I/R injury is the loss of the brush border of proximal tubular cells at apical surfaces.^7^ In our study, we observed a large amount of renal tubular epithelial cell necrosis, resulting in exposure of the basement membrane, proximal tubular dilation and granular cast formation in post‐I/R WT mice. After FXR knockout, we observed obvious reductions in casts and vacuolar degeneration in some tubular epithelial cells, indicating that FXR deficiency could significantly attenuate renal I/R damage. However, our results are in sharp contrast to a previous study by Gai et al^26^ describing a protective role for the FXR agonist 6‐ethyl‐chenodeoxycholic acid (6‐ECDCA) when administered via intraperitoneal injection before renal I/R. One main difference between the two studies was the intervention method of FXR. To our knowledge, 6‐ECDCA is a selective agonist of FXR, which may result in altered effects with different dosages in intestinal I/R, myocardial I/R, hepatocellular carcinoma and diabetes, among others.^11^, ^12^, ^15^, ^27^ Gai et al^26^ also found that oral and intraperitoneal delivery of 6‐ECDCA could produce very different effects. Moreover, 6‐ECDCA might have other pharmacological effects than in FXR signalling to protect against renal I/R. Our result showed that in vitro, FXR‐overexpression plasmid aggravated the apoptosis of renal tubular epithelial cells.

AKI resulting from I/R involves multiple mechanisms, among which immune/inflammatory cells play a key role.^28^, ^29^, ^30^ Our previous study demonstrated that renal macrophages and natural killer T (NKT) cells participate in the mediation of renal I/R injury and recovery.^18^, ^31^ FXR was found to be an important regulator of the inflammatory response.^32^, ^33^ FXR was confirmed to directly interact with NF‐κB. NF‐κB binds to the promoter of FXR and FXR target genes in models of inflammation.^34^, ^35^, ^36^ To determine whether FXR in inflammatory cells was involved in I/R‐mediated injury, we conducted BMT. Deletion of FXR in inflammatory cells did not reduce post‐I/R injury, suggesting that FXR inactivation in inflammatory cells did not influence the final results of renal I/R. Tubular epithelial cells are also important cell types in I/R injury. With high metabolic rates and unique blood flow characteristics, the S3 segment of the proximal tubule is the most common injury site.^7^ Furthermore, injured tubular epithelial cells released proinflammatory cytokines and chemokines, recruiting immune cells, expressing adhesion factors to activate immune cells and promoting an inflammatory response.^29^ In our study, we observed that the number of H/R‐induced apoptotic cells in FXR‐silenced HK2 cells was significantly lower than in control siRNA‐transfected cells. These results, together with the immunohistochemistry staining findings showing FXR expression localization, strongly indicated that I/R injury was mediated by FXR in tubular epithelial cells.

Previous studies have suggested that in renal I/R injury, necrotic cell death is rare and is limited to the outer medullary area, which is highly sensitive to ischaemia and hypoxia, whereas apoptosis is more common in other areas, especially in proximal and distal renal tubular cells.^7^ Consistent with these reports, in our study, after I/R injury, there were more TUNEL‐positive cells in WT than *Fxr^−/−^* mice. H/R induced high levels of cell apoptosis in control siRNA‐transfected HK2 cells, but not in FXR‐silenced HK2 cells. RNA‐seq analysis showed that FXR insufficiency affected apoptosis pathway–related gene expression in post‐I/R kidneys. These results indicated that FXR may activate apoptotic signals in response to ischaemic and hypoxic stress. Several signalling pathways involved in apoptosis, including the endogenous pathway (Bcl‐2 family, cytochrome c and caspase‐9), exogenous pathway (FAS, FADD and caspase‐8) and regulatory pathway (p53 and NF‐κB), were found to be activated in AKI. Cell survival depends on the relative concentrations of proapoptotic molecules (Bax, Bad and Bim) and anti‐apoptotic molecules (Bcl‐2 and Bcl‐2 like protein 1) in the Bcl‐2 family.^21^, ^37^, ^38^ We used a protein array to examine proteins that were possibly involved in the apoptosis signalling pathway in I/R injury. Bad was found to be the most significantly differentially expressed apoptosis‐related protein between WT and *Fxr^−/−^* mice after I/R. We hypothesize that FXR might regulate the expression of Bad in response to I/R injury. Bad is a proapoptotic member of the Bcl‐2 family. Bad promotes apoptosis by binding to anti‐apoptotic proteins, Bcl‐2 and Bcl‐xL, and inhibiting their functions.^38^ Under physiological conditions, Bad can be phosphorylated by the serine‐threonine protein kinase Akt. p‐Bad usually exists in the cytoplasm in an inactive form and cannot induce apoptosis. In contrast, dephosphorylated Bad interacts with Bcl‐2 or Bcl‐xL on the outer membrane of the mitochondria to produce antagonism, leading to the opening of the mitochondrial membrane permeability transition pore (MPTP) and release of cytochrome c.^39^, ^40^ Ohi et al^41^ showed that the maintenance of Bad phosphorylation strongly inhibited sinusoidal endothelial cell apoptosis after liver I/R injury. In the present study, FXR deficiency resulted in an increase in the proportion of phosphorylated Bad both in vivo and in vitro. Moreover, FXR‐deficient kidneys had higher ratios of Bcl‐2 and Bcl‐xL to Bax than WT kidneys after I/R injury. The increase in Bcl‐2 and Bcl‐xl is protective, while the increase in Bax promotes apoptosis. The ratio of Bcl‐2/Bcl‐xL and Bax determines whether apoptosis occurs.^21^ The insufficiency of FXR facilitates Bad phosphorylation, isolating Bad from the Bcl‐2/Bcl‐xL complex and leading to less apoptosis after I/R.

Bad phosphorylation is dependent on the activation of Akt. Akt, also known as protein kinase B, is an anti‐apoptotic protein kinase and is one of the downstream targets of PI3k. PI3K phosphorylates Akt, thus activating Akt. Activated Akt phosphorylates Bad and inhibits the apoptotic function of Bad by sequestering Bad from the Bcl‐2/Bcl‐xL complex.^38^, ^42^ To verify whether FXR is involved in I/R injury by inhibiting the phosphorylation of Bad, we treated both mice and cells with wortmannin, an inhibitor of PI3K. Jang et al^22^ confirmed that wortmannin could reduce Akt and Bad phosphorylation in I/R‐preconditioned kidneys, thus reducing the anti‐apoptotic effect of ischaemic preconditioning. Kaushal et al^43^ also confirmed that PI3K‐mediated phosphorylation of Akt activates Bad phosphorylation, inhibiting cisplatin‐induced caspase‐3 and caspase‐9 activation. In our study, wortmannin treatment reduced Akt and Bad activation as well as the ratio of Bcl‐2/Bcl‐xL and Bax expression in FXR siRNA‐transfected cells. Furthermore, inhibition of Bad prevented the anti‐apoptotic effect conferred by FXR deficiency in both ischaemia and hypoxia and resulted in a loss of the protective effects of FXR deficiency in renal function. These results imply that the increased resistance of *Fxr^−/−^* kidneys against I/R‐induced apoptosis and renal functional impairment is associated with increased Bad phosphorylation.

In conclusion, the present study indicates that FXR insufficiency promotes the Bad phosphorylation‐mediated anti‐apoptotic effect, which has a protective role against renal I/R injury. These findings strongly suggest that FXR could be a therapeutic target for renal I/R injury.

## CONFLICT OF INTEREST

The authors declare no conflicts of interest.

## Supporting information

Figure S1

Figure S2

Figure S3

Figure S4

Figure Legends

## Data Availability

The data that support the findings of this study are available from the corresponding author upon reasonable request.
