# Supplementary figures and images for: Farnesoid X receptor promotes renal ischaemia‐reperfusion injury by inducing tubular epithelial cell apoptosis
Source: Cell Prolif. 2021 Feb 16;54(4):e13005. doi: 10.1111/cpr.13005 (PMC8016637; doi:10.1111/cpr.13005)

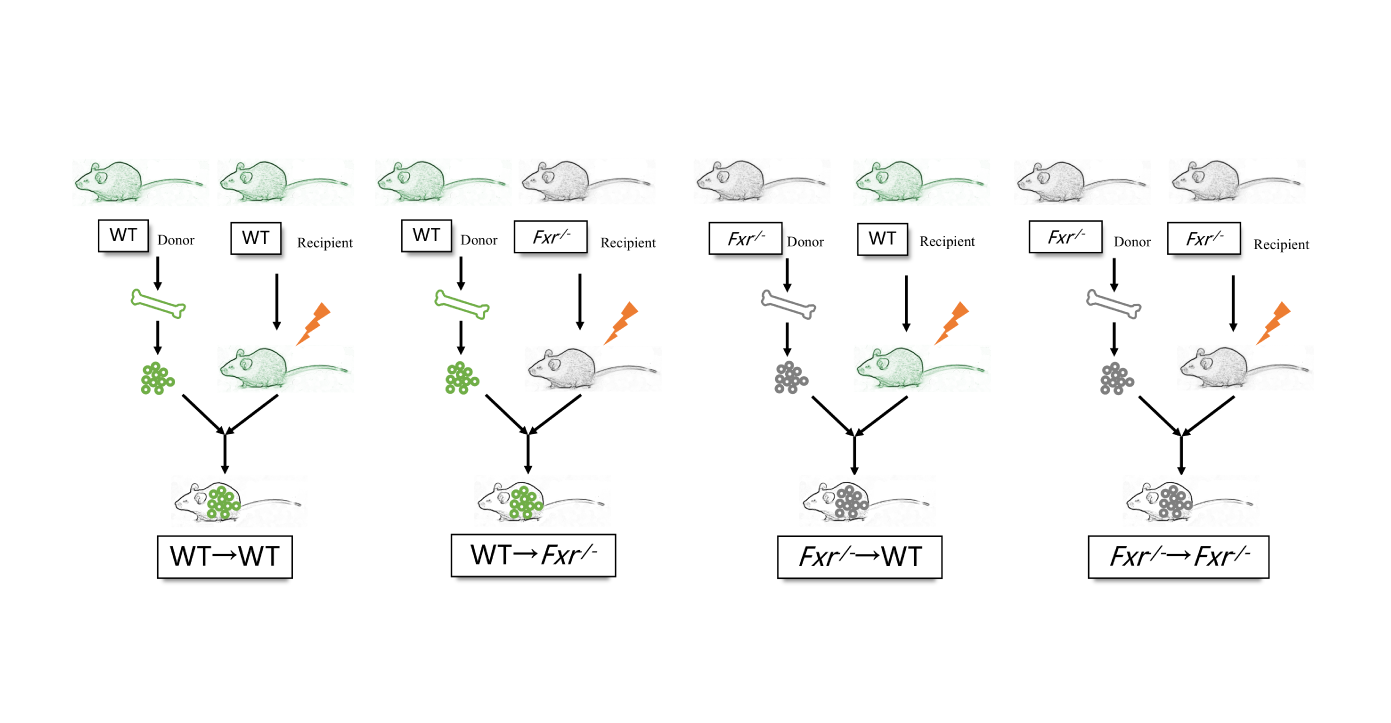

Supplement: Supplementary file 1 — Figure S1 [file CPR-54-e13005-s001.tif]

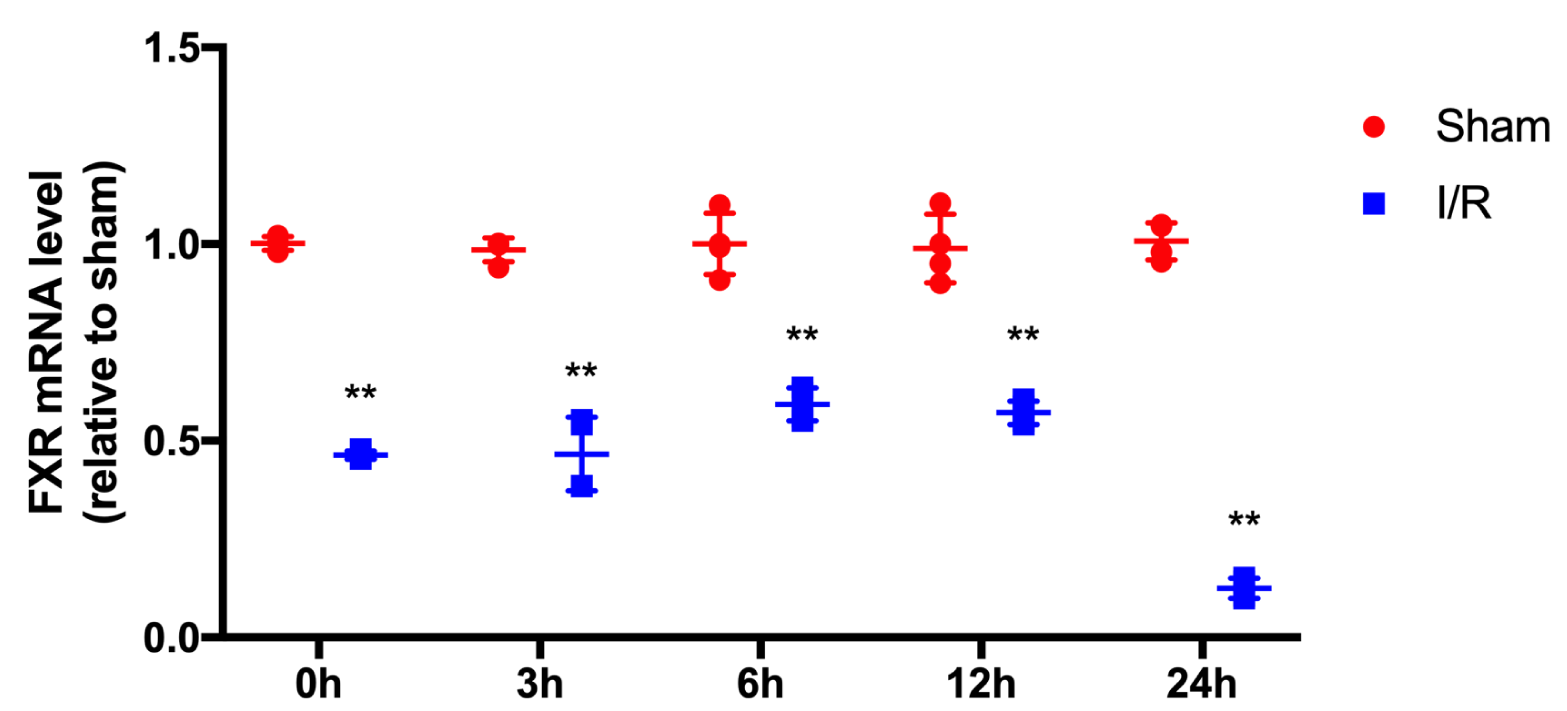

Supplement: Supplementary file 2 — Figure S2 [file CPR-54-e13005-s005.tif]

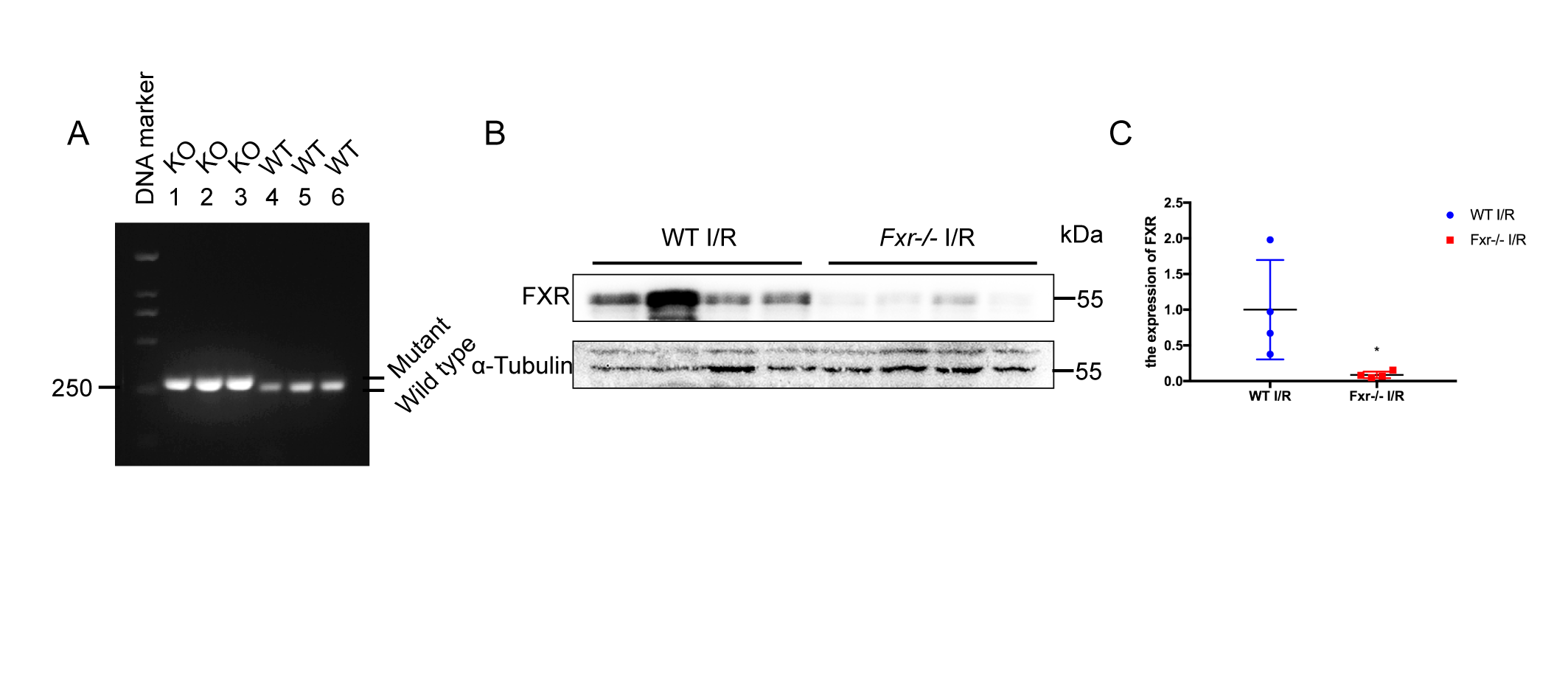

Supplement: Supplementary file 3 — Figure S3 [file CPR-54-e13005-s002.tif]

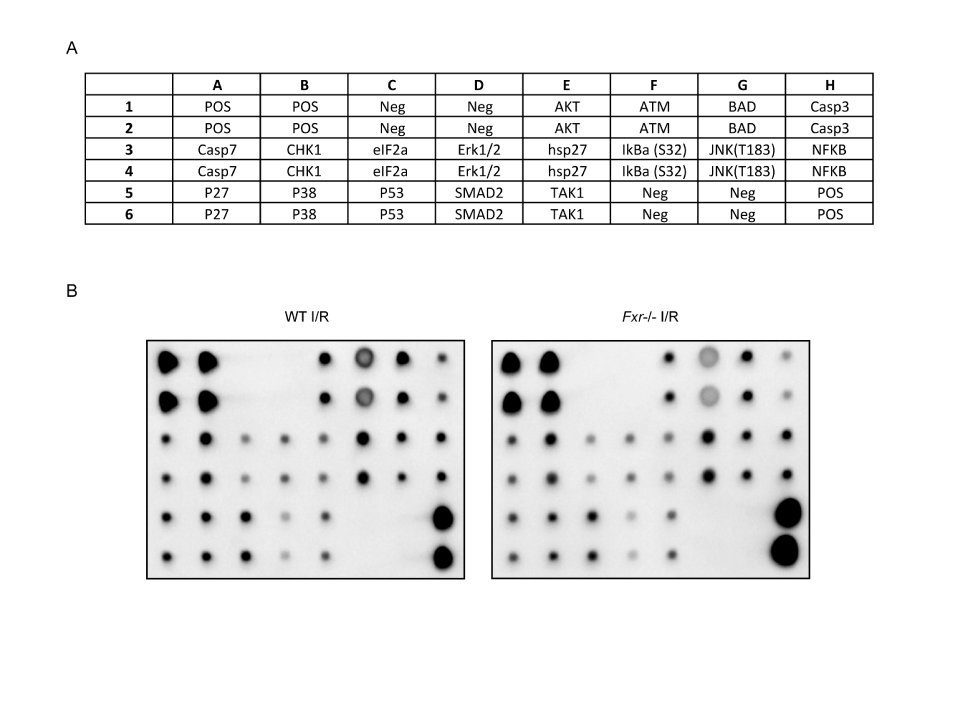

Supplement: Supplementary file 4 — Figure S4 [file CPR-54-e13005-s004.tif]
