# Supplementary material for: Farnesoid X receptor promotes renal ischaemia‐reperfusion injury by inducing tubular epithelial cell apoptosis
Source: Cell Prolif. 2021 Feb 16;54(4):e13005. doi: 10.1111/cpr.13005 (PMC8016637; doi:10.1111/cpr.13005)
Supplement: Supplementary file 5 — Figure Legends [file CPR-54-e13005-s003.docx]

**Supplementary Information**

**Renal I/R model and drug treatment**

A warm renal I/R model was established as described.^1,2^ The operation was performed on a homeothermic operating table (ALC-HTP Homeothermic System, Shanghai Alcott Biotech Co. Ltd, China) to maintain the temperature of the mice at 36±0.2 °C, as detected by a rectal probe. Right nephrectomy was carried out after midline abdominal incision. After the intraperitoneal injection of heparin (50 U/kg), the left renal pedicle was clamped with an atraumatic microvascular clamp for 20 or 25 min. The sham operation group underwent the surgical procedures without vascular occlusion. The operation was conducted by an experienced researcher without prior information about the prior treatment and genetic background of the animals. Animals were sacrificed 24 h after reperfusion to obtain serum and kidney tissues for further analyses.

The phosphatidylinositol 3-kinase (PI3K) inhibitor wortmannin (1 mg/kg, dissolved in DMSO; S2758, Selleck, USA) was intraperitoneally administered 24 h prior to the subsequent I/R or sham operation.

All animal experiments were conducted following the NIH guidelines for the Care and Use of Laboratory Animals and the Animal Protocol Committee of Shanghai Jiaotong University and were approved by the Animal Care Committee at the Renji Hospital, School of Medicine, Shanghai Jiaotong University.

**Cell culture and treatment**

The human proximal tubular cell line (HK-2) was acquired from the American Type Culture Collection (ATCC, Manassas, VA, USA). Cells were cultured according to the suppliers’ instructions. For hypoxia-reoxygenation (H/R) treatment, cells were seeded at a concentration of 5*10^5^ cells/2 ml/well in a 6-well flat-bottomed plate and were cultured overnight to allow cell adhesion. Cells were then exposed to hypoxia (1% O2) for 24 h before being returned to normoxic conditions. Cells were collected at 6 h after reoxygenation. The PI3K inhibitor wortmannin (100 µmol/l, dissolved in DMSO; S2758, Selleck, USA) was added to the culture medium for 48 h.

**Renal function, survival, histology and histomorphological scoring of acute tubular injury**

Plasma creatinine (Cr) and urea nitrogen (BUN) levels were measured with a standard spectrophotometric assay (Roche Diagnostic GmbH, Germany). The Kaplan-Meier survival analytical method was used to estimate the survival rate and to generate a survival curve for the mice. An experienced observer who had no prior information about the treatment and genetic background of the mice evaluated the state of the mice after I/R. If they were morbid, including experiencing serious immobility, a hunchback, hypothermia or no response to noise, death was considered inevitable. In this case, the animal was euthanized by exsanguination under anesthesia.

Kidney tissues were fixed overnight in 10% neutral buffered formalin, dehydrated, embedded in paraffin and sectioned in 4-μm sections. Periodic acid-Schiff (PAS) staining was used for histological analysis. The tubular injury was analyzed in a blinded manner by a nephropathologist based on tubular cell necrosis, tubular dilation, intratubular necrotic debris, and cast formation (original magnification *200). Tubular injury was graded by a semiquantitative histomorphological scoring system from 0 to 5 as follows: 0, none; 1, <10%; 2, 10–25%; 3, 26–45%; 4, 46–75%; and 5, >75%. At least 3 fields were evaluated per section and averaged per slide.

**Polymorphonuclear leukocyte (PMN) infiltration**

Myeloperoxidase (MPO, polyclonal rabbit antibody; NBP1-42591, Novus Biologicals, USA) was used to immunohistochemically localize PMNs and was then visualized with diaminobenzidine (DAB) and counterstained with hematoxylin. The immunohistochemical localization of FXR in renal sections was determined using an NR1H4 antibody (1:200, #A9003A, R&D Systems, USA). The number of infiltrating PMNs was calculated with ImageJ (National Institutes of Health, USA) by two researchers blinded to the experimental conditions.

**Terminal deoxynucleotidyl transferase-mediated 2’ deoxyuridine 5’-triphosphate nick-end labeling assay (TUNEL)**

Apoptotic cells were detected in renal sections with an ApopTag Fluorescein In Situ Apoptosis Detection Kit S7110 (Chemicon International, USA) according to the manufacturer’s protocol. Cells with positive nuclear staining by fluorescent antibodies were visualized by fluorescence microscopy and were counted (original magnification *400), as previously described.^2,3^ At least 3 fields were evaluated per section.

**RNA sequencing (RNA-seq) and the identification of differentially expressed transcripts**

Kidney tissues were sent to the Genminix Biological Company (Shanghai, China) for microarray analysis. Gene expression microarray analyses were performed with Affymetrix GeneChip Mouse transcriptome assays 2.0 (Affymetrix, Santa Clara, USA). The Genminix Biological Company performed the bioinformatics analysis. Microarray data were uploaded to the Gene Expression Omnibus.

**Mice apoptosis proteome profiler array**

To investigate the pathways by which FXR induces apoptosis, we examined apoptosis-related proteins using a proteome profiler array (mouse apoptosis signaling pathway array kit, Raybiotech, Norcross, GA, USA) according to the manufacturer’s protocol. Briefly, kidneys were treated with lysis buffer for 30 min, and 500 μg/ml of protein extract from each sample was incubated with the antibody array membrane for 2 h. The membrane image file was analyzed using an ImageQuant LAS4000 Scanner.

**Bone marrow transplantation (BMT)**

BMT was performed as previously described (Figure 7).^1,3^ Briefly, male recipient mice aged 8–10 weeks were irradiated with a lethal dose of 8.0 Gy (X-ray). Bone marrow cells were harvested from male donors aged 8–10 weeks. Four hours after irradiation, bone marrow cells (5*10^6^) were injected into the tail vein of recipient mice. Renal I/R procedures were conducted 30 days after BMT.

**Western blot (WB) antibodies**

Total protein extracts from kidney tissues were separated by 12% sodium dodecyl sulfate-polyacrylamide gel electrophoresis (SDS- PAGE), transferred to polyvinylidene difluoride membranes, and incubated with primary antibodies overnight. The primary antibodies were as follows: FXR (1:1000, #A9003A, R&D Systems, USA), phosphorylated Bcl-2 agonist of cell death (p-Bad) (1:500, #9291, Cell Signaling Technologies, USA), Bcl-2 agonist of cell death (Bad) (1:1000, #9292, Cell Signaling Technologies, USA), Akt (1:1000, #2920, Cell Signaling Technologies, USA), cleaved caspase-3 (1:1000, #9664, Cell Signaling Technologies, USA), p-PI3K(1:1000, ab86714, Abcam, USA), Bax (1:1000, #2772, Cell Signaling Technologies, USA), Bcl-2 (1:1000, 12789-1-AP, Proteintech, USA), and Bcl-xL (1:1000, #MAB894, R&D Systems, USA);

**Transcriptional analysis**

Total kidney RNA was extracted and assessed by quantitative real-time polymerase chain reaction (RT-PCR) using SYBR Premix Ex Taq (Takara, Japan), and the values were normalized to the β-actin expression values. Primers were purchased from Takara: CCTCGGAACAGAAACCTTGT and TTACAACCGTGGGAGGTGTA (FXR); and CACCATTGGCAATGAGCGGTTC and AGGTCTTTGCGGATGTCCACGT (β-actin).

**Small interfering RNA (siRNA)**

The FXR-specific siRNA sequences were GAAUUCGAAAUAGUGGUAUCUCUGA and UCAGAGAUACCACUYAUUUCGAAUUC. Transfection procedures were performed according to the manufacturer’s protocol. Briefly, cells were transfected at a final siRNA duplex concentration of 20 nM in Opti-Mem (Life Technologies Corporation, NY, USA) by using Lipofectamine RNAiMAX (Life Technologies Corporation, NY, USA) in 6-well culture plates for 6 h. Twenty-four hours after transfection, cells were subjected to H/R treatment.

**Fluorescence-activated cell sorting (FACS) analysis**

Flow cytometry was used to analyze apoptosis after H/R. Briefly, cells were collected, washed and transferred to a 5-ml culture tube. Five microliters of FITC Annexin V and 5 µl propidium iodide (PI) was added from an apoptosis kit (#556547, BD Biosciences, USA), and then the mixture was gently vortexed and incubated for 15 min. Flow cytometry data acquisition was performed on a FACSCalibur (BD Biosciences, USA). Data were analyzed by FlowJo software 9.0 (Tree Star, USA).

**References**

1 He, K. *et al.* Lipopolysaccharide-induced cross-tolerance against renal ischemia-reperfusion injury is mediated by hypoxia-inducible factor-2alpha-regulated nitric oxide production. *Kidney Int* **85**, 276-288, doi:10.1038/ki.2013.342 (2014).

2 Zhang, J. *et al.* Hypoxia-Inducible Factor-2alpha Limits Natural Killer T Cell Cytotoxicity in Renal Ischemia/Reperfusion Injury. *J Am Soc Nephrol* **27**, 92-106, doi:10.1681/ASN.2014121248 (2016).

3 Dong, B. *et al.* Ischemia/reperfusion-induced CHOP expression promotes apoptosis and impairs renal function recovery: the role of acidosis and GPR4. *PLoS One* **9**, e110944, doi:10.1371/journal.pone.0110944 (2014).

**Supplementary Figure Legends**

Figure S1. Bone marrow transplantation (BMT). Male recipient mice aged 8–10 weeks were irradiated with a lethal dose of 8.0 Gy (X-ray). Bone marrow cells were harvested from male donors aged 8–10 weeks. Four hours after irradiation, bone marrow cells (5*10^6^) were injected into the tail vein of recipient mice.

Figure S2. Time courses of FXR mRNA expression in the renal after I/R were measured by RT-PCR. Total RNA was extracted from kidney tissues at 0 h, 3 h, 6 h, 12 h and 24 h after reperfusion, respectively. Each plotted as fold change relative to mRNA levels in heart (A) or at 0 h after I/R (B).

Figure S3. Confirmation of the successful generation of FXR-knockout mice. (A) Polymerase chain reaction (PCR) genotyping of *Fxr^-/-^* mice (mutant, 291bp; wild type, 249bp). (B) Representative Western blot (WB) images of FXR in renal tissue at 24 h post-I/R (n=4 per group). (C) Expression of FXR at 24 h post-I/R. The densities of FXR protein bands were quantified with ImageJ analytical software and normalized to α-tubulin. Each column represents the mean ± SD. *P<0.05 vs. wild-type mice at the same time point after I/R injury.

Figure S4. Quantitative analysis of the mouse apoptosis signaling pathway array in post-I/R kidney. Kidneys were lysed at 24-h reperfusion after 20-min ischemia and total tissue protein was extracted. (A) The exact protein name of each dot in the array. (B) Representative images of the apoptotic protein array are shown for the wild-type mice(left), *Fxr^-/-^* mice (right).
